# A behavior-based drug screening system using a *Caenorhabditis elegans* model of motor neuron disease

**DOI:** 10.1038/s41598-019-46642-6

**Published:** 2019-07-12

**Authors:** Kensuke Ikenaka, Yuki Tsukada, Andrew C. Giles, Tadamasa Arai, Yasuhito Nakadera, Shunji Nakano, Kaori Kawai, Hideki Mochizuki, Masahisa Katsuno, Gen Sobue, Ikue Mori

**Affiliations:** 1 Department of Neurology, Nagoya University Graduate School of Medicine, Nagoya, Japan; 2 Group of Molecular Neurobiology, Graduate School of Science, Nagoya University, Nagoya, Japan; 3 Neuroscience Institute, Graduate School of Science, Nagoya University, Nagoya, Japan; 4 Department of Neuroscience, The Scripps Research Institute, Scripps Florida, Jupiter, Florida USA; 5 Pharmaceutical Research Laboratories, Toray Industries, Inc., Kamakura, Kanagawa Japan; 6 Department of Neurology, Osaka University Graduate School of Medicine, Suita, Osaka, Japan; 7 Brain and Mind Research Center, Nagoya University, Nagoya, Aichi Japan

**Keywords:** Cell death in the nervous system, Amyotrophic lateral sclerosis

## Abstract

Amyotrophic lateral sclerosis (ALS) is a fatal neurodegenerative disease characterized by the progressive loss of motor neurons, for which there is no effective treatment. Previously, we generated a *Caenorhabditis elegans* model of ALS, in which the expression of *dnc-1*, the homologous gene of human *dynactin-1*, is knocked down (KD) specifically in motor neurons. This *dnc-1* KD model showed progressive motor defects together with axonal and neuronal degeneration, as observed in ALS patients. In the present study, we established a behavior-based, automated, and quantitative drug screening system using this *dnc-1* KD model together with Multi-Worm Tracker (MWT), and tested whether 38 candidate neuroprotective compounds could improve the mobility of the *dnc-1* KD animals. We found that 12 compounds, including riluzole, which is an approved medication for ALS patients, ameliorated the phenotype of the *dnc-1* KD animals. Nifedipine, a calcium channel blocker, most robustly ameliorated the motor deficits as well as axonal degeneration of *dnc-1* KD animals. Nifedipine also ameliorated the motor defects of other motor neuronal degeneration models of *C*. *elegans*, including *dnc-1* mutants and human TAR DNA-binding protein of 43 kDa overexpressing worms. Our results indicate that *dnc-1* KD in *C*. *elegans* is a useful model for the screening of drugs against motor neuron degeneration, and that MWT is a powerful tool for the behavior-based screening of drugs.

## Introduction

Amyotrophic lateral sclerosis (ALS) is a fatal neurodegenerative disease characterized by the progressive loss of motor neurons. Approximately 5–10% of ALS cases are familial, whereas approximately 90% are sporadic (SALS). To understand the pathological mechanisms of SALS and to develop effective drugs, we previously characterized the motor neuron-specific gene expression profile of SALS patients^1,2^ Among the dysregulated genes, *dynactin-1*was markedly downregulated in motor neurons from an early disease stage. Dynactin-1 is a crucial component of dynactin, which is a multiprotein complex associated with dynein^3^, the molecular motor for retrograde transport^4^. Interestingly, missense mutations in *dynactin-1* are linked to familial lower motor neuron disease^5^, or Perry syndrome, a familial type of Parkinson disease involving TAR DNA-binding protein of 43 kDa (TDP-43) aggregation^6^. Recently, it has been demonstrated that Perry syndrome patients have diverse symptoms similar to those observed in Parkinson’s disease and a type of motor neuron disease that involves frontotemporal degeneration^7^. On the other hand, it was also reported that the loss of TDP-43 impaired the fusion of autophagosomes with lysosomes through the downregulation of *dynactin-1*, leading to the accumulation of immature autophagic vesicles^8^. These reports indicate the bidirectional regulation between TDP-43 pathology and *dynactin-1* downregulation, which is a good candidate to explain the pathogenesis of SALS.

To analyze the effect of *dynactin-1* downregulation in motor neuron degeneration, we generated a *Caenorhabditis elegans* (*C*. *elegans*) model of motor neuron disease, in which the expression of *dnc-1*, the homologous gene of human *dynactin-1*, is knocked down specifically in motor neurons^9^. This model shows progressive motor defects together with axonal and neuronal degeneration. Pathologically, we also observed axonal spheroids, degenerated mitochondria, ubiquitin-positive inclusions, and an increased number of autophagosomes in degenerated neurons, reflecting the pathology of SALS motor neurons.

In the present study, we used our new *C*. *elegans* model and established a behavioral screening assay using an automated tracking system, the Multi-Worm Tracker (MWT)^10^. Using this assay, we assessed 38 compounds from 5 categories: (1) approved by the FDA for ALS treatment, (2) approved by the FDA for other treatments and under or past clinical trials for ALS, (3) approved by the FDA for other treatments and found to have promising effects on ALS models, (4) autophagy activators based on our previous finding that rapamycin, an autophagy activator, improves the motor defects of a *dnc-1* KD model^9^, and (5) histone deacetylase (HDAC) inhibitors based on our previous finding that trichostatin A, an HDAC inhibitor, improves the motor deficits of the *dnc-1* KD model by activating tubulin acetylation and enhancing axonal transport^9^. Of the 38 compounds, we found that 12 compounds, including riluzole, the drug internationally approved for ALS, improved the motor deficits of *dnc-1* KD animals. Among those 12 compounds, we found that nifedipine, a calcium-channel blocker, was the most effective drug for improving the *dnc-1* KD motor defect. Follow-up experiments showed that nifedipine also ameliorated the neurodegeneration of motor neurons in *dnc-1* KD animals.

## Results

### Adapting MWT for the screening of pharmacological effects on *C*. *elegans* locomotion

**Figure 1:**
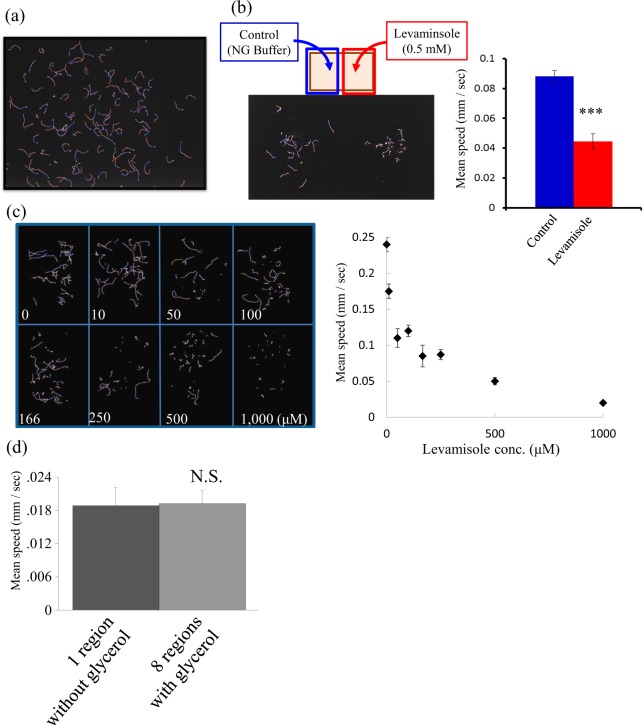
Optimization of the drug-screening system using Multi-Worm Tracker (MWT) (**a**) A representative trajectory of an N2 worm for 30 sec. A 13 × 10 cm agar-filled plate was used for the assay, and images were captured using Toshiba-Teli Ultra-High-resolution 12 M pixel CMOS sensor camera-link camera. The locomotion of as much as about 500 worms can be detected and analyzed simultaneously. (**b**) Performance of the MWT assay for detection of levamisole treatment. Control worms treated with NG buffer only were placed on the left side of the assay plate (blue line, n = 18) and levamisole (0.5 mM) on the right side (red line n = 20). The average moving speed of each group was analyzed and quantified using Choreography. (**c**) The WMT assay is divided into 8 groups. To analyze multiple groups of worms simultaneously, assay plates were divided into 8 regions by glycerol. A representative trajectory of worms treated by different concentrations of levamisole is shown. The average moving speeds analyzed by Choreography are shown on the right side. (**d**) A comparison of the locomotion speed of the worms with and without separation into regions by glycerol The statistical analyses in b and d were performed using the Student *t*-test (***p < 0.0001).

MWT was designed to quantify the behavior of many worms simultaneously on a 5-cm diameter Petri dish^10^. For the purpose of drug screening, we adapted the MWT to record a larger field of view using a higher resolution camera (12 Mpixels) and a larger rectangular assay plate (4 cm × 10 cm × 1.45 cm). This modified system enables us to analyze more animals at once (~500 animals) without increasing the population density (Fig. 1a). Thus, the assay plate can be divided into regions to test multiple treatments at once (i.e., Fig. 1b, 2 regions of ~250 worms each; Fig. 1c, 8 regions of ~40 worms each). Regions were separated by glycerol because worms avoid the high osmolarity of glycerol and will not cross between regions^11^. We confirmed that the separation into 8 compartments by glycerol did not change the moving speed of the animals compared to that without separation (Fig. 1d).

**Figure 2:**
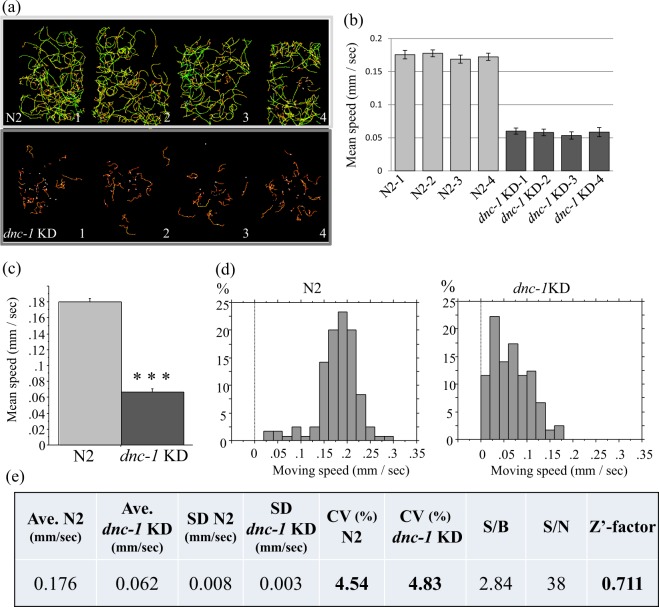
Evaluation of *dnc-1* KD worms as a model for drug screening by MWT. (**a**) Representative trajectories of N2 (upper four groups) and *dnc-1* KD worms (lower four groups) analyzed in the same assay plate by WMT. Moving speeds are depicted as a linear rainbow-color gradient. (**b**) The average moving speeds of each region (N2-1 to 4: light gray bars; *dnc-1* KD-1 to 4: dark gray bars). (**c**) The average moving speed of N2 and *dnc-1* KD worms (N2, n = 120; *dnc-1* KD worms, n = 121). (**d**) Representative histogram of moving speeds of N2 and *dnc-1* KD worms. (**e**) Statistical analysis and evaluation of *dnc-1* KD worms for the drug screening by MWT. The Z’-factor is defined as: Z’-factor = 1–3(SD_N2 + SD_*dnc-1* KD)/(Ave._N2-Ave._*dnc-1*KD). The statistical analysis in C was performed using the Student *t*-test (***p < 0.0001).

We validated the effectiveness of the adapted system by analyzing whether this system could detect drug-induced motor defects. For this purpose, we used levamisole, a nicotinic acetylcholine receptor agonist, which causes muscle paralysis in *C*. *elegans*, and quantified the locomotor speed of levamisole-treated animals. We detected a dose-dependent, unequivocal motor defect caused by levamisole (Fig. 1b,c), showing that the system developed in this study was effective as a behavior-based drug-screening system.

### MWT efficiently detected the motor defect in *dnc-1* KD worms

Previously, we analyzed the motor function of *dnc-1* KD worms by the bending assay and thrashing assay, in which we manually scored the number of body bends of worms on an agar plate or in liquid, respectively. *dnc-1* KD worms showed a 60–70% reduction in mobility compared with wild-type worms^9^.

We analyzed the effects of *dnc-1* KD on locomotion using MWT (Fig. 2a–d). Consistent with the previous assays, the speed of *dnc-1* KD worms was reduced by 70%. The variations in speed within a region and between regions were very small (Fig. 2c), suggesting that each region can be used to test a different treatment during a drug screen. Additionally, in contrast to the several hours it took for screening in previous methods, conducting the MWT assay and analyzing the results only took 15 min, thereby making it more feasible to conduct a screen to find drugs that improve the *dnc-1* KD motor defect (Fig. 2e).

### Autophagy activation attenuated the motor defect of *dnc-1* KD worms

**Figure 3:**
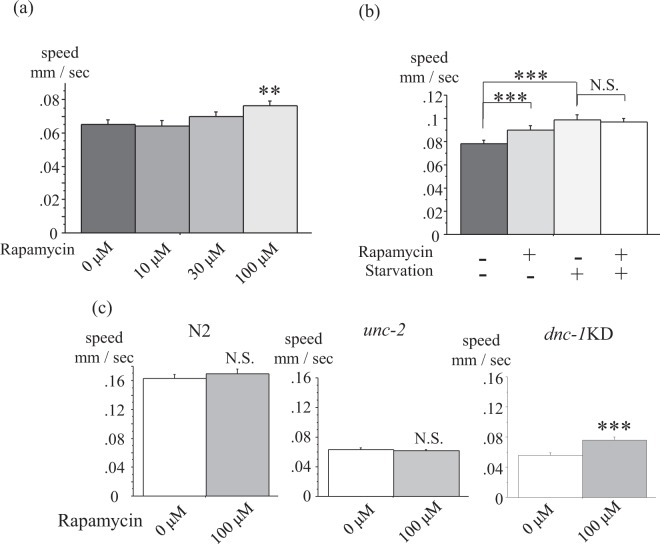
MWT detected the significant and specific effect of rapamycin on locomotion in *dnc-1* KD worms. (**a**) Dose-dependent effects of rapamycin on locomotion in *dnc-1* KD worms (n = 166, 120, 179, and 164 for rapamycin 0, 10, 30, and 100 μM, respectively). (**b**) Effects of single or combination therapy of rapamycin and starvation to *dnc-1* KD worms (n = 248, 125, 67, and 70 for control, rapamycin only, starvation only, and a combination of both therapies, respectively). (**c**) The specificity of rapamycin to *dnc-1* KD worms. No significant effect of rapamycin was observed in N2 and *unc-2* mutant worms (n = 46 and 30 for control and rapamycin to N2, respectively; n = 20 and 35 for control and rapamycin to *unc-2*, respectively; and n = 124 and 93 for control and rapamycin to *dnc-1* KD worms, respectively). Statistical analyses were performed by one-way ANOVA followed by the Bonferroni/Dunn post hoc test (**a**,**b**) and the Student *t*-test (**c**) (**p < 0.001 and ***p < 0.0001). Error bars indicate the S.E.M.

Autophagy activation by rapamycin or food restriction (starvation) ameliorates the motor defect of *dnc-1* KD worms by attenuating axonal degeneration^9^. We hence tested whether MWT could detect the effect of rapamycin and starvation in *dnc-1* KD worms. We confirmed the dose-dependent effect of rapamycin (Fig. 3a), and also showed that starvation had a similar effect to rapamycin without synergy (Fig. 3b).

To verify the specificity of the effect of autophagy activation, we compared the effect of rapamycin between wild-type, *unc-2* mutants, and *dnc-1* KD worms (Fig. 3c). *unc-2* encodes a *C*. *elegans* homologue of a voltage-gated calcium channel and its mutation causes a severe motor defect^12^. Although the *dnc-1* KD worms were markedly affected by rapamycin, wild-type and *unc-2* mutant worms did not show a significant change by rapamycin, indicating the specific effect of rapamycin on *dnc-1* KD worms (Fig. 3c). Thus, we used rapamycin as a positive control for our drug screen to find drugs that improve the motor defects of *dnc-1* KD worms.

### Blinded screening of 38 compounds by MWT found that riluzole, an ALS drug, improved the motor defect of *dnc-1* KD worms

**Figure 4:**
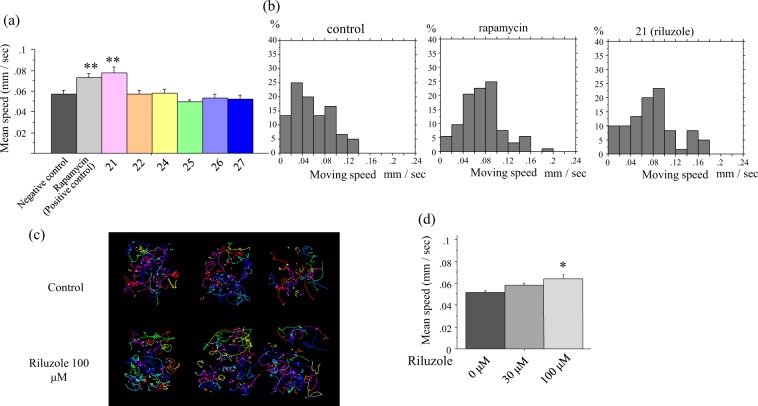
MWT identified riluzole, a drug widely used for ALS treatment, as a positive hit compound. (**a**) Results of the MWT assay from one screening plate. Rapamycin is a positive control and the other compounds were tested in a blind manner on *dnc-*1 KD worms and analyzed by MWT. Note: drug 21 is riluzole. (**b**) Histograms of the moving speed of control-, rapamycin-, and riluzole-treated *dnc-1* KD worms, showing a right shift in rapamycin- and riluzole-treated groups. (**c**) Representative trajectories of control worms (upper three maps) and riluzole-treated worms (lower three maps). (**d**) Dose-dependent effect of riluzole on locomotion of *dnc-1* KD worms. Statistical analyses were performed by one-way ANOVA followed by the Bonferroni/Dunn post hoc test (**a**,**d**) (*p < 0.05, and **p < 0.001). Error bars are S.E.M.

Using the automated behavior-based drug-screening system, we assessed in a blinded manner whether any of the 38 compounds, which have been reported or were expected to have a neuroprotective effect, could improve the motor defects of *dnc-1* KD worms. Rapamycin and dimethyl sulfoxide (DMSO) were included as positive and negative controls, respectively. The results of the screening are listed in Table 1. We found that 12 compounds were as effective as or more effective than rapamycin. Among these 12 compounds, riluzole, the drug internationally approved for ALS, improved the locomotion speed of *dnc-1* KD worms (Fig. 4). In Fig. 4a, we present a representative result of an assay, showing that among six compounds, only no. 21 (riluzole) ameliorated the speed of *dnc-1* KD worms. The histograms of rapamycin-treated and riluzole-treated worms show a right shift compared with the negative control, demonstrating that these treatments improved the speed of *dnc-1* KD worms (Fig. 4b). The tracks of control and riluzole-treated *dnc-1* KD worms during the assay are also shown in Fig. 4c. We also found that the effect of riluzole is dose-dependent (Fig. 4d). Given that riluzole is an approved medication for ALS patients and was identified using the MWT automated behavior-based drug screen for *dnc-1* KD worms, other compounds identified by the screen are potential candidates that may be useful for the treatment for ALS.

**Table 1:** Result of drug screening by Multi Worm Tracker.

	Compound name	Blind number	Compound source	Mechanism of action	Highest phase for ALS treatment	MWT results (ratio to non treated *dnc-1* KD worms)
1	Nifedipine	15	FUJIFILM Wako Pure Chemical Corporation	Calcium channel blocker	Approved for other diseases	1.69
2	Masitinib	14	AK Scientific	Tyrosine kinase inhibitor	P-III	1.34
3	WN1316	2	Synthetic sample in lab	NAIP enhancer	—	1.32
4	BYL-719	28	MedChemExpress	PI3Kα inhibitor	P-III in other diseases	1.32
5	Riluzole	21	LKT Labs	Glutamate release inhibitor	Approved	1.32
6	Tubastatin A	30	MedChemExpress	HDAC6 inhibitor	—	1.29
7	GSK-2606414	5	MedChemExpress	PERK inhibitor	Preclinical study	1.29
8	Retinoic acid	16	FUJIFILM Wako Pure Chemical Corporation	—	Preclinical study	1.29
9	VORINOSTAT	29	MedChemExpress	HDAC Inhibitor (HDACs 1, 2, 3, 6)	—	1.29
10	SA-4503	10	MedChemExpress	σ1 receptor agonist	Clinical trial	1.24
11	CK-2017357	13	ChemShuttle	Troponin activator	Preclinical study	1.24
12	Rasagiline	7	Sigma-Aldrich	MAO-B Inhibitor	Clinical trial	1.23
13	Rapamycin	Positive control	Tokyo Chemical Industry	mTOR inhibitor	Preclinical study	1.22
14	AICAR	34	Tokyo Chemical Industry	AMPK activator	—	1.19
15	Bromhexine	17	FUJIFILM Wako Pure Chemical Corporation	—	Preclinical study	1.17
16	—	3	Synthetic sample in lab	EPHA4 inhibitor	Preclinical study	1.15
17	ACY-1215	31	MedChemExpress	HDAC6 inhibitor	—	1.14
18	METFORMIN	33	FUJIFILM Wako Pure Chemical Corporation	AMPK activator	—	1.12
19	AVex-73	20	Synthetic sample in lab	Acts on M1 muscarinic sodium channels Receptor Agonist	Preclinical study	1.12
20	Tamoxifen	33	FUJIFILM Wako Pure Chemical Corporation	Estrogen receptor modulator	—	1.09
21	D-(+)-Trehalose	36	Tokyo Chemical Industry	Autophagy modulator	—	1.09
22	Methylcobalamin	23	FUJIFILM Wako Pure Chemical Corporation	—	Clinical trial	1.07
23	SB-431542	6	MedChemExpress	ALK5 Inhibitor	Preclinical study	1.07
24	Dasatinib	11	MedChemExpress	Bcr-Abl inhibitor, tyrosine kinase inhibitor	Preclinical study	1.06
25	—	4	Synthetic sample in lab	EPHA4 inhibitor	Preclinical study	1.05
26	Kenpaullone	1	LKT Labs	GSK-3β inhibitor CDK inhibitor	Preclinical study	1.03
27	—	19	Synthetic sample in lab	mSOD1 aggregation inhibitor	Preclinical study	1.01
28	Fluoxetine	38	Tokyo Chemical Industry	Serotonin transporter inhibitor	—	1.00
29	SPAUTIN-1	35	Sigma-Aldrich	Autophagy modulator	—	0.98
30	Edaravone	22	Tokyo Chemical Industry	Free radical scavenger	Approved	0.98
31	—	18	Synthetic sample in lab	P2X7 antagonist	Preclinical study	0.97
32	Pyrimethamine	9	Sigma-Aldrich	Dihydrofolate reductase inhibitor	Clinical trial	0.95
33	Arimoclomol	12	SEQUOIA	Heat-shock protein 70inducer	Clinical trial	0.95
34	Fingolimod	37	Tokyo Chemical Industry	S1P receptor agonist	Clinical trial	0.94
35	Deforolimus	26	MedChemExpress	mTOR inhibitor	—	0.92
36	Ibudilast	8	FUJIFILM Wako Pure Chemical Corporation	Phosphodiesterase PDE4 inhibitor	Clinical trial	0.91
37	AZD-8055	24	MedChemExpress	mTOR inhibitor	—	0.88
38	PF-04691502	27	MedChemExpress	mTOR inhibitor PI3K inhibitor	—	0.84
39	PP242	25	Sigma-Aldrich	mTOR inhibitor	—	0.83

### L-type calcium blocker nifedipine ameliorated the motor defects and axonal degeneration of *dnc-1* KD worms

**Figure 5:**
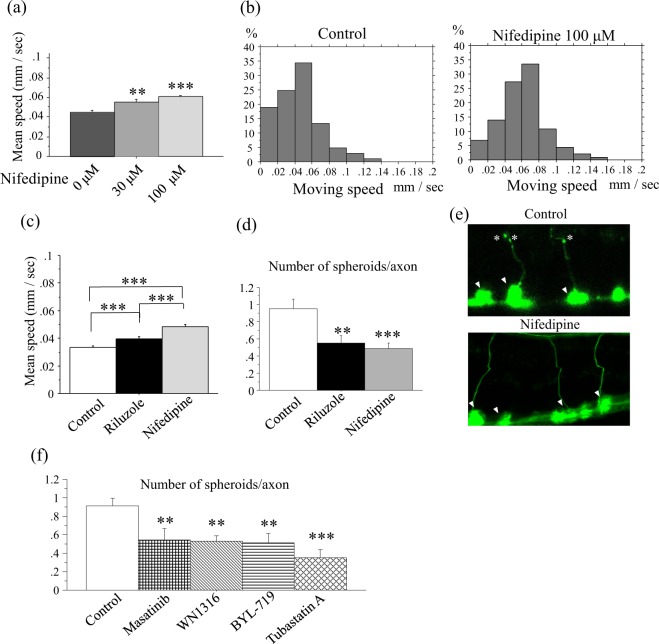
Effects of nifedipine on the locomotion and axonal pathology of *dnc-*1 KD worms. (**a**) Dose-dependent effects of nifedipine on the locomotion of worms (n = 205, 170, and 230 for 0, 30, and 100 μM of nifedipine, respectively). (**b**) Histograms of the moving speed of control and nifedipine-treated *dnc-1* KD worms. (**c**) Direct comparison of the effect of riluzole and nifedipine (n = 180, 210, and 170 for control, riluzole, and nifedipine). (**d**,**e**) The effects of riluzole and nifedipine on the pathology of *dnc-*1 KD worms. The number of axonal spheroids per transverse axon was counted after treatment by each compound (n = 20 worms for each group) (**d**), and representative images of a transverse axon with and without treatment by nifedipine are shown (**e**). Asterisks are axonal spheroids and arrowheads are the cell body of ventral motor neurons (**e**). (**f**) Number of axonal spheroids in a transverse axon treated by the hit compounds of the MWT assay (n = 20 worms for each group). Statistical analyses were performed by one-way ANOVA followed by the Bonferroni/Dunn post hoc test (**a**,**c**,**d**,**f**). (*p < 0.05, **p < 0.001, and ***p < 0.0001). Error bars indicate the S.E.M.

During the blinded screen, we found four compounds that were as effective as or more effective than riluzole (Table 1). Nifedipine, an L-type calcium channel blocker, showed the strongest effect on the locomotion of *dnc-1* KD worms. Just as in the case of riluzole, the effect of nifedipine was dose-dependent (Fig. 5a). The distribution of the speed of nifedipine-treated *dnc-1* KD worms was clearly shifted to the right compared with control-treated *dnc-1* KD worms (median speed = 0.043 mm/sec and 0.061 mm/sec; DMSO-treated group and nifedipine-treated group, respectively), suggesting that nifedipine uniformly improves the motor function of *dnc-1* KD worms (Fig. 5b). We also directly compared the effects of riluzole and nifedipine, and found that nifedipine had a stronger effect on the locomotion of *dnc-1* KD worms than riluzole (Fig. 5c).

The motor defect in *dnc-1* KD worms is caused by the degeneration of motor neurons^9^. Therefore, we tested whether nifedipine can improve the motor neuron degeneration in *dnc-1* KD worms. In *dnc-1* KD animals, axonal spheroids is a key feature of neurodegeneration, showing the accumulation of damaged mitochondria and autophagosomes^9^. We scored a number of axonal spheroids in a transverse section of ventral motor neuron axons (Fig. 5d), and found that riluzole and nifedipine significantly ameliorated the axonal degeneration in *dnc-1* KD worms.

We also tested the effects of the remaining 4 of the top 6 compounds from MWT screening by scoring axonal spheroids. All 4 compounds significantly decreased the number of axonal spheroids in *dnc-1* KD animals (Fig. 5f).

**Figure 6:**
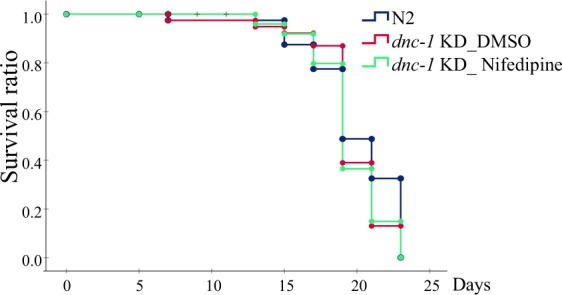
Lifespan analysis of nifedipine-treated animals. Kaplan meier curves of the wild type (N2) (n = 80) and *dnc-1* KD animals treated with DMSO (n = 80) or 100 μM of nifedipine (n = 80). There were no significant differences between all groups according to the log-rank test.

Given that in the *dnc-1* KD model, *dnc-1* shRNA is expressed under the ventral motor-neuron specific *acr-2* promoter, other neurons and organs, including head neurons, and gut and pharyngeal muscles, which are essential for living, are intact in the *dnc-1* KD model. Consistently, lifespan analysis showed no significant changes among DMSO-treated wild-type, DMSO-treated *dnc-1* KD, and nifedipine-treated *dnc-1* KD worms (Fig. 6).

### Nifedipine ameliorated the motor defects in other worm models with motor neuron degeneration

**Figure 7:**
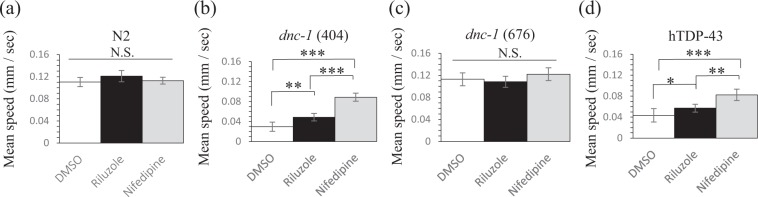
Effects of riluzole and nifedipine on other models of motor neuron degeneration. Each worm line was treated with DMSO, riluzole, or nifedipine and then assayed by MWT. Statistical analyses were performed by one-way ANOVA followed by the Bonferroni/Dunn post hoc test. (*p < 0.05, **p < 0.001, and ***p < 0.0001). Error bars indicate the S.E.M.

We further investigated the effects of riluzole and nifedipine on other worm models showing motor neuron degeneration and motor defects, to analyze the specificity and generality of these compounds. Both drugs did not show an increase in locomotion speed in wild-type worms (Fig. 7a). To evaluate the effect on *dnc-1* mutants, we tested two alleles, *dnc-1* (*or404ts*) and *dnc-1* (*or676ts*), both of which show microtubule-associated defects in a temperature-dependent manner^13,14^. In our assay condition at 20 °C, only *dnc-1* (*or404ts*) showed slow locomotion, and the drugs only affected the locomotion of *dnc-1* (*or404ts*) (Fig. 7b,c). We also observed that both drugs ameliorated locomotor defects of human TDP-43-overexpressing strains (Fig. 7d). This strain expresses human wild-type TDP-43 under the pan-neuronal promotor, *snb-1*, and shows motor neuronal degeneration and progressive locomotor defects^15^. Importantly, the effect of nifedipine was significantly stronger than riluzole in *dnc-1* KD animals, *dnc-1* (*or404ts*) mutants, and hTDP-43 overexpressing animals, indicating the potential of nifedipine as a new candidate compound for the amelioration of motor neuron degeneration.

## Discussion

In this study, we developed a behavior-based drug screening system by combining a *C*. *elegans* model of ALS-like motor degeneration and dysfunction with an adapted version of the MWT. Our new screening system can evaluate eight compounds at once and only takes 15 min. We confirmed that the assay can detect the motor defects of *dnc-1* KD worms, as well as the effects of rapamycin and food restriction on this *dnc-1* KD model^9^.

We used this novel assay system to blindly test the effects of 38 compounds on *dnc-1* KD worms. One of the most crucial results was that riluzole improved the motor defects of *dnc-1* KD worms. As riluzole is an internationally approved drug for ALS, it is an indication that compounds identified by this approach will be worth pursuing as potential treatments for ALS. As we used compounds that have been previously reported or expected to have neuroprotective effects (see the criteria for selection in the Materials and Methods section), many compounds (25 out of 38) improved the motor phenotypes of *dnc-1* KD worms. Among these compounds, we found that nifedipine, an L-type calcium-channel blocker, had the strongest effect on the locomotion of the worms.

Following the locomotor assay, we investigated the effects of the compounds on axonal degeneration. Importantly, all the top 6 compounds identified by the MWT assay had a significant effect on axonal degeneration. Moreover, we confirmed that nifedipine ameliorated the motor defects of other models of motor neuron disease, *dnc-1* mutant animals, and human TDP-43 transgenic animals.

These results suggest that the adapted MWT assay can be a powerful system to perform behavior-based screening of drugs against motor neuron degeneration. The limitation of this assay is its throughputness. Several groups have developed worm-tracking systems to analyze many worms at once^16–19^. However, the application of these systems to drug screening in a high-throughput manner has been extremely challenging. Although our system, which enables the simultaneous analysis of 8 drugs using 500 worms, is the most powerful screening system to our knowledge to date, we still need faster and simpler screening systems for screening tens of thousands of drugs. Shunmoogum *et al*. performed a screening of 3,850 small molecules using mutant TDP-43-overexpressing *C*. *elegans*^20^. In their initial screening, they visually evaluated the motility of the worms and then confirmed the reproducibility and specificity of the results using a quantitative method, by manually scoring the percentage of paralyzed worms^20^. Considering that the big advantages of our system are its accuracy, reproducibility, and quantitativity, our newly established system will be a very strong tool as a second-throughput screening.

## Materials and Methods

### *C. elegans* culture

Standard methods were used to culture *C*. *elegans* on nematode growth medium (NGM) agar seeded with OP50 *Escherichia coli* (*E*. *coli*)^21^. The worms were maintained at 20 °C unless otherwise indicated.

### *C. elegans* strains

The following strains were used in this study: N2 wild-type (Bristol), SBG8 Ex[P*acr-2*::EmGFP::*dnc-1*miRNA#1; P*gcy-8*::GFP]^9^, CB55 *unc-2 (e55)*^22^, EU1006 *dnc-1 (or404)*^13^, EU1257 *dnc-1 (or676)*^14^, and CL6049 dvIs62 [snb-1p::hTDP-43/3′ long UTR + mtl-2p::GFP], which were obtained from the *C*. *elegans* Genetics Center.

### Compound selection

The compounds selected were as follows: (1) approved by the FDA for ALS treatment, (2) currently under/or finished clinical trials for ALS, and (3) show promising effects on ALS models. We also analyzed some of autophagy activators and HDAC inhibitors as positive controls because in a previous study we found that rapamycin, an autophagy activator, and trichostatin A, an HDAC inhibitor, were effective on this model^9^.

### Synchronization of worms and compound treatment in liquid culture

Twenty gravid adult *dnc-1* KD animals were selected by their uncoordinated phenotype and allowed to lay eggs on NGM plates with OP50 *E*. *coli* for 3 h to obtain approximately 200 synchronized eggs. Plates were cultivated for 3 days until the eggs had developed into adults. For the culture of temperature-sensitive *dnc-1* mutant lines, they were incubated on NGM plates for 5 days at 15 °C until they had developed into the same age. Then worms were transferred into liquid medium (S basal medium with concentrated OP50 [80 mg/mL]) with compounds dissolved in DMSO at a final concentration of 100 μM (1% DMSO). We used drugs at 100 μM according to the most effective concentration of the positive control, rapamycin^9^. Two-hundred μL of the liquid culture (~50 worms) was transferred into each well of a 48-well plate with a flat bottom and without coating (Falcon/351178). The source of the compounds is listed in Table 1. The plate was incubated with shaking at 100 rpm for 16 h in 20 °C.

For levamisole treatment, approximately 300 synchronized adult worms (day 4) were collected from NGM plates and divided into 24-well plates with different concentrations of levamisole hydrochloride (Sigma-Aldrich, USA) (0, 10, 20, 50, 100, 200, 400, and 1,000 μM) dissolved in M9 buffer. After incubation for 10 minutes in 24-well plates, worms were transferred to the assay plate and analyzed by MWT.

### Locomotion assay by MWT

After the incubation with a compound, worms were washed 3 times in NG buffer and gently transferred onto an assay plate. The assay plate was a 13 × 10-cm plate filled with agar, which was divided into 8 regions of equal area. Regions were surrounded with glycerol, an aversive stimulus for *C*. *elegans*, to keep animals from moving over to the other regions. Filter paper (Whatman/3MM paper) was used to remove excess NG buffer. Worms from a given compound-treated group were placed in 1 of the 8 regions with various compounds being tested simultaneously.

An adapted version of the MWT^10^ was used to record the locomotion of *C*. *elegans* on the agar plate. Adaptations included a Toshiba-Teli Ultra High Resolution 12 M pixel CMOS sensor camera-link camera (CSC12M25BMP19-01B), a lens (RICHO FL-YFL3528), and an adaptor (Toshiba-Teli FTAR-2). In addition, experiments were performed under dark-field lighting conditions using the ring LED light (CCS Inc. LDR-206SW2-LA1). *C*. *elegans* locomotion was recorded for 10 mins.

### Analysis of the MWT data

Analysis of the recordings was performed using Choreography (part of the MWT software) and custom-written scripts to organize and summarize the data. Animal tracks were collected as a time series of the centroid position for each frame of the final 2 minutes of the recording. We used the final 2 minutes to enable animals to first adapt to the circumstances of the assay and to perform animal recognition by the tracker plateau. This was particularly important for slow-moving animals because the tracker only identifies animals that have moved from their initial position. The following Choreography filters were used to avoid image artifacts: ‒shadowless and -t 10. The speed of an individual animal was calculated as the sum of distances between sequential centroids divided by the duration of the track. Experimental groups were summarized using mean and standard errors of the mean, weighted by the duration of an animal’s track.

### Microscopic analysis

Microscopic analysis of worms was performed as previously described^9^. Briefly, the worms were anesthetized by placing them in an 8-μL drop of levamisole (2 mM) on solidified pads of 2% agarose laid on slides with a coverslip. Worms were observed using a confocal microscope (Zeiss LSM 710).

For the evaluation of axonal degeneration, we scored the number of axonal spheroids per transverse axon.

### Lifespan assay

A lifespan assay was performed as described previously^13^, with some modifications. Synchronized 3 day-old worms were collected and 20 worms were transferred to each NGM plate containing 100 μM 5-fluoro-2′-deoxyuridine (Sigma-Aldrich, USA), 100 μM drug (control or nifedipine), and 0.1% DMSO. For each group, 80 worms were transferred every 4 days to a freshly prepared plate. The animals were scored as dead if they did not move when prodded with a platinum pick and did not show pharyngeal pumping.

### Statistical analysis

Statistical analyses were performed using StatView software version 5 (Hulinks, Tokyo, Japan). The Student *t*-test was used for the comparison of two independent groups and one-way analysis of the variance (ANOVA) with the Bonferroni/Dunn post-hoc test for more than three groups. We used the Kaplan-Meier and log-rank test to compare survivals of N2 and *dnc-1* KD worms with our without treatment by riluzole or nifedipine. The application of these methods are indicated in each figure and legend.
